# On the Higher Power Sums of Reciprocal Higher-Order Sequences

**DOI:** 10.1155/2014/521358

**Published:** 2014-03-10

**Authors:** Zhengang Wu, Jin Zhang

**Affiliations:** ^1^Department of Mathematics, Northwest University, Xi'an, Shaanxi, China; ^2^School of Mathematics and Computer Engineering, Xi'an University of Arts and Science, Xi'an, Shaanxi, China

## Abstract

Let {*u*_*n*_} be a higher-order linear recursive sequence. In this paper, we use the properties of error estimation and the analytic method to study the reciprocal sums of higher power of higher-order sequences. Then we establish several new and interesting identities relating to the infinite and finite sums.

## 1. Introduction

The so-called Fibonacci zeta function and Lucas zeta function, defined by

(1) $$\begin{array}{c} \zeta_{F}\left(s\right)=\overset{\infty }{\underset{n=1}{\sum }}\frac{1}{F_{n}^{s}},\;\;\zeta_{L}\left(s\right)=\overset{\infty }{\underset{n=1}{\sum }}\frac{1}{L_{n}^{s}}, \end{array}$$

where the *F*_*n*_ and *L*_*n*_ denote the Fibonacci numbers and Lucas numbers, have been considered in several different ways; see [1, 2]. Ohtsuka and Nakamura [3] studied the partial infinite sums of reciprocal Fibonacci numbers and proved the following conclusions:

(2) $$\begin{array}{c} \left\lfloor {\left(\begin{array}{cc} \overset{\infty }{\underset{k=n}{\sum }} & \frac{1}{F_{k}} \end{array}\right)}^{-1}\right\rfloor =\{\begin{array}{cc} F_{n-2} & \text{if}\;n\;\text{is}\;\text{even},n\geq 2,\\ F_{n-2}-1 & \text{if}\;n\;\text{is}\;\text{odd},n\geq 1, \end{array}\\ \left\lfloor {\left(\begin{array}{cc} \overset{\infty }{\underset{k=n}{\sum }} & \frac{1}{F_{k}^{2}} \end{array}\right)}^{-1}\right\rfloor =\{\begin{array}{cc} F_{n-1}F_{n}-1 & \text{if}\;n\;\text{is}\;\text{even},n\geq 2,\\ F_{n-1}F_{n} & \text{if}\;n\;\text{is}\;\text{odd},n\geq 1, \end{array} \end{array}$$

where ⌊·⌋ denotes the floor function.

Further, Wu and Zhang [4, 5] generalized these identities to the Fibonacci polynomials and Lucas polynomials. Various properties of the Fibonacci polynomials and Lucas polynomials have been studied by many authors; see [6–13].

Recently, some authors considered the nearest integer of the sums of reciprocal Fibonacci numbers and other well-known sequences and obtained several meaningful results; see [14–16]. In particular, in [16], Kılıç and Arıkan studied a problem which is a little different from that of [3], namely, that of determining the nearest integer to (∑_*k*=*n*_^*∞*^(1/*v*_*k*_))^−1^. Specifically, suppose that ||*x*|| = ⌊*x* + (1/2)⌋ (the nearest integer function) and {*v*_*n*_}_*n*≥0_ is an integer sequence satisfying the recurrence formula

(3) $$\begin{array}{c} v_{n}=pv_{n-1}+qv_{n-2}+v_{n-3}+\cdots +v_{n-k}, \end{array}$$

for any positive integer *p* ≥ *q* and *n* > *k*. Then we can conclude that there exists a positive integer *n*_0_ such that

(4) $$\begin{array}{c} ||{\left(\begin{array}{cc} \overset{\infty }{\underset{k=n}{\sum }} & \frac{1}{v_{k}} \end{array}\right)}^{-1}||=v_{n}-v_{n-1}, \end{array}$$

for all *n* > *n*_0_.

In [17], Wu and Zhang unified the above results by proving the following conclusion that includes all the results, [3–8, 15, 16], as special cases.

**Proposition 1**

For any positive integer *n* > *m*, the *m*th-order linear recursive sequence {*u*_*n*_} is defined as follows:

(5) $$\begin{array}{c} u_{n}=a_{1}u_{n-1}+a_{2}u_{n-2}+\cdots +a_{m-1}u_{n-m+1}+a_{m}u_{n-m}, \end{array}$$

with initial values *u*_*i*_ ∈ *ℕ* for 0 ≤ *i* < *m* and at least one of them not being zero. For any positive real number *β* > 2 and any positive integer *a*_1_ ≥ *a*_2_ ≥ ⋯≥*a*_*m*_ ≥ 1, there exists a positive integer *n*_1_ such that

(6) $$\begin{array}{c} ||{\left(\begin{array}{cc} \overset{\left\lfloor \beta n\right\rfloor }{\underset{k=n}{\sum }} & \frac{1}{u_{k}} \end{array}\right)}^{-1}||=u_{n}-u_{n-1},\;\left(n\geq n_{1}\right). \end{array}$$

In particular, taking *β* → +*∞*, there exists a positive integer *n*_2_ such that

(7) $$\begin{array}{c} ||{\left(\overset{\infty }{\underset{k=n}{\sum }}\frac{1}{u_{k}}\right)}^{-1}||=u_{n}-u_{n-1},\;\left(n\geq n_{2}\right). \end{array}$$

It seems difficult to deal with (∑_*k*=*n*_^*∞*^(1/*u*_*k*_^*s*^))^−1^ for all integers *s* ≥ 2, because it is quite unclear* a priori* what the shape of the result might be. In [18], Xu and Wang applied the method of undetermined coefficients and constructed a number of delicate inequalities in order to study the infinite sum of the cubes of reciprocal Pell numbers and then obtained the following meaningful result.

**Proposition 2**

For any positive integer *n* ≥ 1, we have the identity

(8) ⌊(∑k=n∞1Pk3)−1⌋ ={Pn2Pn−1+3PnPn−12+⌊−6182Pn−9182Pn−1⌋        if  n  is  even, n≥2,Pn2Pn−1+3PnPn−12+⌊6182Pn+9182Pn−1⌋        if  n  is  odd, n≥1.

To find and prove this result is a substantial achievement since such a complex formula would not be clear beforehand that a result would even be possible. However, there is no research considering the higher power (*s* > 2) of reciprocal sums of some recursive sequences. The main purpose of this paper is using the properties of error estimation and the analytic method to study the higher power of the reciprocal sums of {*u*_*n*_} and obtain several new and interesting identities. The results are as follows.

**Theorem 3**

Let {*u*_*n*_} be an *m*th-order sequence defined by (5) with the restrictions *a*_1_, *a*_2_,…, *a*_*m*_ ∈ *ℕ* and *a*_1_ ≥ *a*_2_ ≥ ⋯≥*a*_*m*_ ≥ 2. For any real number *β* > 2 and positive integer 1 ≤ *s* < ⌊log⁡_(*α*/*a*_1_)_ *αd*⌋, where *α*, *α*_1_,…, *α*_*m*−1_ are the roots of the characteristic equation of *u*_*n*_ and *d* ^−1^ = max⁡ {|*α*_1_|, |*α*_2_|,…, |*α*_*m*−1_|}, then there exists a positive integer *n*_3_ such that

(9) $$\begin{array}{c} ||{\left(\begin{array}{cc} \overset{\left\lfloor \beta n\right\rfloor }{\underset{k=n}{\sum }} & \frac{a_{1}^{sk}}{u_{k}^{s}} \end{array}\right)}^{-1}-\left(\frac{u_{n}^{s}}{a_{1}^{sn}}-\frac{u_{n-1}^{s}}{a_{1}^{sn-s}}\right)||=0,\;\left(n\geq n_{3}\right). \end{array}$$

Taking *β* → +*∞*, from Theorem 3 we may immediately deduce the following.

**Corollary 4**

Let {*u*_*n*_} be an *m*th-order sequence defined by (5) with the restrictions *a*_1_, *a*_2_,…, *a*_*m*_ ∈ *ℕ* and *a*_1_ ≥ *a*_2_ ≥ ⋯≥*a*_*m*_ ≥ 2. For positive integer 1 ≤ *s* < ⌊log⁡_(*α*/*a*_1_)_ *αd*⌋, where *α*, *α*_1_,…, *α*_*m*−1_ are the roots of the characteristic equation of *u*_*n*_ and *d* ^−1^ = max⁡{|*α*_1_ | , |*α*_2_ | ,…, |*α*_*m*−1_|}, then there exists a positive integer *n*_4_ such that

(10) $$\begin{array}{c} ||{\left(\begin{array}{cc} \overset{\infty }{\underset{k=n}{\sum }} & \frac{a_{1}^{sk}}{u_{k}^{s}} \end{array}\right)}^{-1}-\left(\frac{u_{n}^{s}}{a_{1}^{sn}}-\frac{u_{n-1}^{s}}{a_{1}^{sn-s}}\right)||=0,\;\left(n\geq n_{4}\right). \end{array}$$

For positive real number 1 < *β* ≤ 2, whether there exits an identity for

(11) $$\begin{array}{c} ||{\left(\begin{array}{cc} \overset{\left\lfloor \beta n\right\rfloor }{\underset{k=n}{\sum }} & \frac{a_{1}^{sk}}{u_{k}^{s}} \end{array}\right)}^{-1}|| \end{array}$$

is an interesting open problem.

## 2. Several Lemmas 

To complete the proof of our theorem, we need two lemmas.

**Lemma 5**

Let *a*_1_, *a*_2_,…, *a*_*m*_ ∈ *ℕ* with *a*_1_ ≥ *a*_2_ ≥ ⋯≥*a*_*m*_ ≥ 1 and *m* ∈ *ℕ* with *m* ≥ 2. Then for the polynomial

(12) $$\begin{array}{c} f\left(x\right)=x^{m}-a_{1}x^{m-1}-a_{2}x^{m-2}-\cdots -a_{m-1}x-a_{m}, \end{array}$$

we have the following:

(I) polynomial *f*(*x*) has exactly one positive real zero *α* with *a*_1_ < *α* < *a*_1_ + 1;
(II) other *m* − 1 zeros of *f*(*x*) lie within the unit circle in the complex plane.

**Proof**

See Lemma 1 of [16].

**Lemma 6**

Let *m* ≥ 2 and {*u*_*n*_}_*n*≥0_ be an integer sequence satisfying the recurrence formula (5). Then for any positive integer *s*, we have

(13) $$\begin{array}{c} u_{n}^{s}=c^{s}\alpha^{sn}+O\left(\alpha^{sn-n}d^{-n}\right)\;\left(n⟶\infty \right), \end{array}$$

where *c* > 0, *d* > 1, and *a*_1_ < *α* < *a*_1_ + 1 is the positive real zero of *f*(*x*).

**Proof**

From Lemma 2 of [16], the closed formula of *u*_*n*_ is given by

(14) $$\begin{array}{c} u_{n}=c\alpha^{n}+O\left(d^{-n}\right)\;\left(n⟶\infty \right), \end{array}$$

where *c* > 0, *d* > 1, *a*_1_ < *α* < *a*_1_ + 1, and *α* is the positive real zero of *f*(*x*). Now we prove Lemma 6 by mathematical induction. From formula (14), we have

(15) $$\begin{array}{c} u_{n}^{2}=c^{2}\alpha^{2n}+O\left(d^{-2n}\right)+O\left(\alpha^{n}d^{-n}\right)=c^{2}\alpha^{2n}+O\left(\alpha^{n}d^{-n}\right). \end{array}$$

That is, the lemma holds for *s* = 2. Suppose that for all integers 2 ≤ *s* ≤ *k* we have

(16) $$\begin{array}{c} u_{n}^{s}=c^{s}\alpha^{sn}+O\left(\alpha^{sn-n}d^{-n}\right)\;\left(n⟶\infty \right). \end{array}$$

Then for *s* = *k* + 1 we have

(17) unk+1=(ckαkn+O(αkn−nd−n))·(cαn+O(d−n))=ck+1αkn+n+O(αknd−n) +O(αknd−n)+O(αkn−nd−2n)=ck+1α(k+1)n+O(αknd−n).

That is, Lemma 6 also holds for *s* = *k* + 1. This completes the proof of Lemma 6 by mathematical induction.

## 3. Proof of Theorem 3

In this section, we shall complete the proof of Theorem 3. From the geometric series as *ϵ* → 0, we have

(18) $$\begin{array}{c} \frac{1}{1\pm \epsilon }=1\mp \epsilon +O\left(\epsilon^{2}\right)=1+O\left(\epsilon \right). \end{array}$$

Using Lemma 6, we have

(19) a1skuks=a1skcsαsk+O(αsk−kd−k)=a1skcsαsk(1+O(α−kd−k))=a1skcsαsk(1+O(α−kd−k))=a1skcsαsk+O(a1skαsk+kdk).

Consequently,

(20) ∑k=n⌊βn⌋a1skuks=1cs∑k=n⌊βn⌋(a1α)sk+O(∑k=n⌊βn⌋a1skαsk+kdk)=αscs(αs−a1s)·(a1α)sn−1cs(αs−a1s)·(a1α)s⌊βn⌋ +O(a1snαsn+ndn)=αscs(αs−a1s)·(a1α)sn+O(a1snαsn·a1s⌊βn⌋−snαs⌊βn⌋−sn) +O(a1snαsn·1αndn)=αscs(αs−a1s)·(a1α)sn+O(a1snαsn·h),

where *h* = max⁡ {(*a*_1_^*s*⌊*βn*⌋−*sn*^/*α* ^*s*⌊*βn*⌋−*sn*^), (1/*α* ^*n*^ *d* ^*n*^)}.

Taking the reciprocal of this expression yields

(21) (∑k=n⌊βn⌋a1skuks)−1 =(1)×(αscs(αs−a1s)·(a1α)sn     ·(1+O(a1snhαsn·αsna1sn)))−1 =cs(αs−a1s)αs·(αa1)sn·(1+O(h)) =csαsna1sn−csαsn−sa1sn−s+O(αsna1sn·h) =unsa1sn−un−1sa1sn−s+O(αsna1sn·h).

**Case 1**

If *h* = (*a*_1_^*s*⌊*βn*⌋−*sn*^/*α* ^*s*⌊*βn*⌋−*sn*^), then for any real number *β* > 2 and positive integer *s* we have

(22) $$\begin{array}{c} \frac{\alpha^{sn}}{a_{1}^{sn}}\cdot h=\frac{\alpha^{sn}}{a_{1}^{sn}}\cdot \frac{a_{1}^{s\lfloor \beta n\rfloor -sn}}{\alpha^{s\lfloor \beta n\rfloor -sn}}={\left(\frac{a_{1}}{\alpha }\right)}^{s\lfloor \beta n\rfloor -2sn}<1. \end{array}$$

**Case 2**

If *h* = (1/*α* ^*n*^ *d* ^*n*^), for any positive integer *a*_1_ ≥ 2, 1 < (*α*/*a*_1_) < *αd* holds. Then for any positive integer *s* with

(23) $$\begin{array}{c} 1\leq s<\left\lfloor \log_{\left(\alpha /a_{1}\right)}\alpha d\right\rfloor , \end{array}$$

we have

(24) $$\begin{array}{c} \frac{\alpha^{sn}}{a_{1}^{sn}}\cdot h=\frac{\alpha^{sn-n}}{a_{1}^{sn}d^{n}}={\left(\frac{\alpha^{s-1}}{a_{1}^{s}d}\right)}^{n}<1. \end{array}$$

In both cases, it follows that for any real number *β* > 2 and positive integer 1 ≤ *s* < ⌊log⁡_(*α*/*a*_1_)_ *αd*⌋ there exists *n* ≥ *n*_3_ sufficiently large so that the modulus of the last error term of identity (21) becomes less than 1/2. This completes the proof of Theorem 3.

**Proof of Corollary 4**

From identity (19), we have

(25) $$\begin{array}{c} \frac{a_{1}^{sk}}{u_{k}^{s}}=\frac{a_{1}^{sk}}{c^{s}\alpha^{sk}}+O\left(\frac{a_{1}^{sk}}{\alpha^{sk+k}d^{k}}\right). \end{array}$$

Consequently,

(26) ∑k=n∞a1skuks=1cs∑k=n∞(a1α)sk+O(∑k=n∞a1skαsk+kdk)=αscs(αs−a1s)·(a1α)sn+O(a1snαsn+ndn).

Taking the reciprocal of this expression yields

(27) (∑k=n∞a1skuks)−1 =1(αs/cs(αs−a1s))·(a1/α)sn·(1+O(1/αndn)) =cs(αs−a1s)αs·(αa1)sn·(1+O(1αndn)) =csαsna1sn−csαsn−sa1sn−s+O(αsn−na1sndn) =unsa1sn−un−1sa1sn−s+O(αsn−na1sndn).

For any positive integer *s* with

(28) $$\begin{array}{c} 1\leq s<\left\lfloor \log_{\left(\alpha /a_{1}\right)}\alpha d\right\rfloor , \end{array}$$

we have

(29) $$\begin{array}{c} \frac{\alpha^{sn-n}}{a_{1}^{sn}d^{n}}={\left(\frac{\alpha^{s-1}}{a_{1}^{s}d}\right)}^{n}<1. \end{array}$$

So there exists *n* ≥ *n*_4_ sufficiently large so that the modulus of the last error term of identity (27) becomes less than 1/2. This completes the proof of Corollary 4.

## 4. Computation

We can determine the power *s* of different sequence *u*_*n*_ by MATHEMATICA as the following examples.

**Example 7**

Let *u*_*n*_ be the second-order linear recursive sequence (see Table 1).

**Example 8**

Let *u*_*n*_ be the third-order linear recursive sequence (see Table 2).

**Example 9**

Let *u*_*n*_ be the fifth-order linear recursive sequence (see Table 3).

Therefore, we may immediately deduce the following corollaries.

**Corollary 10**

Let *P*_*n*_ = 2*P*_*n*−1_ + *P*_*n*−2_ be the Pell numbers. For any real number *β* > 2 and positive integer 1 ≤ *s* < 9, there exists a positive integer *n*_5_ such that

(30) $$\begin{array}{c} ||{\left(\overset{\left\lfloor \beta n\right\rfloor }{\underset{k=n}{\sum }}\frac{2^{sk}}{P_{k}^{s}}\right)}^{-1}-\left(\frac{P_{n}^{s}}{2^{sn}}-\frac{P_{n-1}^{s}}{2^{sn-s}}\right)||=0,\;\left(n\geq n_{5}\right). \end{array}$$

**Corollary 11**

Let *T*_*n*_ = 4*T*_*n*−1_ + 3*T*_*n*−2_ + 2*T*_*n*−3_ be the generalized Tribonacci numbers. For any real number *β* > 2 and positive integer 1 ≤ *s* < 11, there exists a positive integer *n*_6_ such that

(31) $$\begin{array}{c} ||{\left(\overset{\left\lfloor \beta n\right\rfloor }{\underset{k=n}{\sum }}\frac{4^{sk}}{T_{k}^{s}}\right)}^{-1}-\left(\frac{T_{n}^{s}}{4^{sn}}-\frac{T_{n-1}^{s}}{4^{sn-s}}\right)||=0,\;\left(n\geq n_{6}\right). \end{array}$$

**Corollary 12**

Let *u*_*n*_ = 5*u*_*n*−1_ + 3*u*_*n*−2_ + 2*u*_*n*−3_ + *u*_*n*−4_ + *u*_*n*−5_ be a fifth-order sequence. For any real number *β* > 2 and positive integer 1 ≤ *s* < 18, there exists a positive integer *n*_7_ such that

(32) $$\begin{array}{c} ||{\left(\overset{\left\lfloor \beta n\right\rfloor }{\underset{k=n}{\sum }}\frac{5^{sk}}{u_{k}^{s}}\right)}^{-1}-\left(\frac{u_{n}^{s}}{5^{sn}}-\frac{u_{n-1}^{s}}{5^{sn-s}}\right)||=0,\;\left(n\geq n_{7}\right). \end{array}$$

## 5. Related Results

The following results are obtained similarly.

**Theorem 13**

Let {*u*_*n*_} be an *m*th-order sequence defined by (5) with the restrictions *a*_1_, *a*_2_,…, *a*_*m*_ ∈ *ℕ* and *a*_1_ ≥ *a*_2_ ≥ ⋯≥*a*_*m*_ ≥ 2. Let *p* and *q* be positive integers with 0 ≤ *q* < *p*. For any real number *β* > 2 and positive integer 1 ≤ *s* < ⌊log⁡_(*α*/*a*_1_)_ *α* ^*p*^ *d* ^*p*^⌋, where *α*, *α*_1_,…, *α*_*m*−1_ are the roots of the characteristic equation of *u*_*n*_ and *d* ^−1^ = max⁡ {|*α*_1_|, |*α*_2_|,…, |*α*_*m*−1_|}, then there exist positive integers *n*_8_, *n*_9_, and *n*_10_ depending on *a*_1_, *a*_2_,…, and *a*_*m*_ such that the following hold.

(a) ||(∑_*k*=*n*_^⌊*βn*⌋^((−*a*_1_)^*sk*^/*u*_*k*_^*s*^))^−1^ − (−1)^*sn*^(*u*_*n*_^*s*^/*a*_1_^*sn*^ + *u*_*n*−1_^*s*^/*a*_1_^*sn*−*s*^)|| = 0, (*n* ≥ *n*_8_).
(b) ||(∑_*k*=*n*_^⌊*βn*⌋^(*a*_1_^*spk*+*sq*^/*u*_*pk*+*q*_^*s*^))^−1^ − (*u*_*pn*+*q*_^*s*^/*a*_1_^*spn*+*sq*^ − *u*_*pn*−*p*+*q*_^*s*^/*a*_1_^*spn*+*sq*−*sp*^)|| = 0, (*n* ≥ *n*_9_).
(c) ||(∑_*k*=*n*_^⌊*βn*⌋^((−*a*_1_)^*spk*+*sq*^/*u*_*pk*+*q*_^*s*^))^−1^ − (−1)^*spn*+*sq*^(*u*_*pn*+*q*_^*s*^/*a*_1_^*spn*+*sq*^ + *u*_*pn*−*p*+*q*_^*s*^/*a*_1_^*spn*+*sq*−*sp*^)|| = 0, (*n* ≥ *n*_10_).

For *β* → +*∞*, we deduce the following identity of infinite sum as special case of Theorem 13.

**Corollary 14**

Let {*u*_*n*_} be an *m*th-order sequence defined by (5) with the restrictions *a*_1_, *a*_2_,…, *a*_*m*_ ∈ *ℕ* and *a*_1_ ≥ *a*_2_ ≥ ⋯≥*a*_*m*_ ≥ 2. Let *p* and *q* be positive integers with 0 ≤ *q* < *p*. For any positive integer 1 ≤ *s* < ⌊log⁡_(*α*/*a*_1_)_ *αd*⌋, where *α*, *α*_1_,…, *α*_*m*−1_ are the roots of the characteristic equation of *u*_*n*_ and *d* ^−1^ = max⁡ {|*α*_1_|, |*α*_2_|,…, |*α*_*m*−1_|}, then there exist positive integers *n*_11_, *n*_12_, and *n*_13_ depending on *a*_1_, *a*_2_,…, and *a*_*m*_ such that the following hold.

(d) ||(∑_*k*=*n*_^*∞*^((−*a*_1_)^*sk*^/*u*_*k*_^*s*^))^−1^ − (−1)^*sn*^(*u*_*n*_^*s*^/*a*_1_^*sn*^ + *u*_*n*−1_^*s*^/*a*_1_^*sn*−*s*^)|| = 0, (*n* ≥ *n*_11_).
(e) ||(∑_*k*=*n*_^*∞*^(*a*_1_^*spk*+*sq*^/*u*_*pk*+*q*_^*s*^))^−1^ − (*u*_*pn*+*q*_^*s*^/*a*_1_^*spn*+*sq*^ − *u*_*pn*−*p*+*q*_^*s*^/*a*_1_^*spn*+*sq*−*sp*^)|| = 0, (*n* ≥ *n*_12_).
(f) ||(∑_*k*=*n*_^*∞*^((−*a*_1_)^*spk*+*sq*^/*u*_*pk*+*q*_^*s*^))^−1^ − (−1)^*spn*+*sq*^(*u*_*pn*+*q*_^*s*^/*a*_1_^*spn*+*sq*^ + *u*_*pn*−*p*+*q*_^*s*^/*a*_1_^*spn*+*sq*−*sp*^)|| = 0, (*n* ≥ *n*_13_).

**Proof**

We shall prove only (c) in Theorem 13 and other identities are proved similarly. From identity (19), we have

(33) $$\begin{array}{c} \frac{{\left(-a_{1}\right)}^{spk+sq}}{u_{pk+q}^{s}}=\frac{{\left(-a_{1}\right)}^{spk+sq}}{c^{s}\alpha^{spk+sq}}\left(1+O\left(\alpha^{-pk-q}d^{-pk-q}\right)\right). \end{array}$$

Consequently,

(34) ∑k=n⌊βn⌋(−a1)spk+squspk+sqs =1cs∑k=n⌊βn⌋(−a1α)spk+sq+O(∑k=n⌊βn⌋a1spk+sqαspk+sq+pk+qdpk+q) =(−1)spn+sq·αscs(αs−a1s)·(a1α)spn+sq−(−1)spn+sqcs(αs−a1s)  ·(a1α)sp⌊βn⌋+sq+O(a1spnαspn+pndpn) =(−1)spn+sq·αscs(αs−a1s)·(a1α)spn+sq+O(a1spnαspn·a1sp⌊βn⌋−spnαsp⌊βn⌋−spn)  +O(a1spnαspn·1αpndpn) =(−1)spn+sq·αscs(αs−a1s)·(a1α)spn+sq+O(a1spnαspn·hp),

where *h* = max⁡ {(*a*_1_^*s*⌊*βn*⌋−*sn*^/*α* ^*s*⌊*βn*⌋−*sn*^), (1/*α* ^*n*^ *d* ^*n*^)}.

Taking the reciprocal of this expression yields

(35) (∑k=n⌊βn⌋(−a1)spk+squpk+qs)−1 =(−1)spn+sq·cs(αs−a1s)αs·(αa1)spn+sq·(1+O(hp)) =upn+qsa1spn+sq+upn−p+qsa1spn+sq−sp+O(αspna1spn·hp).

*Case  1. *If *h* = (*a*_1_^*s*⌊*βn*⌋−*sn*^)/(*α* ^*s*⌊*βn*⌋−*sn*^), then for any real number *β* > 2 and positive integer *s* we have

(36) $$\begin{array}{c} \frac{\alpha^{sn}}{a_{1}^{sn}}\cdot h^{p}=\frac{\alpha^{sn}}{a_{1}^{sn}}\cdot \frac{a_{1}^{sp\lfloor \beta n\rfloor -spn}}{\alpha^{sp\lfloor \beta n\rfloor -spn}}={\left(\frac{a_{1}}{\alpha }\right)}^{sp\lfloor \beta n\rfloor -spn-sn}<1. \end{array}$$

*Case  2. *If *h* = (1/*α* ^*n*^ *d* ^*n*^), for any positive integer *a*_1_ ≥ 2, 1 < (*α*/*a*_1_) < *αd* holds. Then for any positive integer *s* with

(37) $$\begin{array}{c} 1\leq s<\left\lfloor \log_{\left(\alpha /a_{1}\right)}\alpha^{p}d^{p}\right\rfloor , \end{array}$$

we have

(38) $$\begin{array}{c} \frac{\alpha^{sn}}{a_{1}^{sn}}\cdot h^{p}=\frac{\alpha^{sn-pn}}{a_{1}^{sn}d^{pn}}={\left(\frac{\alpha^{s-p}}{a_{1}^{s}d^{p}}\right)}^{n}<1. \end{array}$$

In both cases, it follows that for any real number *β* > 2 and positive integer 1 ≤ *s* < ⌊log⁡_(*α*/*a*_1_)_ *α* ^*p*^ *d* ^*p*^⌋, there exists *n* ≥ *n*_10_ sufficiently large so that the modulus of the last error term of identity (35) becomes less than 1/2. This completes the proof of Theorem 13(c).

## Figures and Tables

**Table 1:** 

*u*_*n*_	*α*	*d*	log⁡_*α*/*a*_1__ *αd*
*u*_*n*_ = 2*u*_*n*−1_ + *u*_*n*−2_	2.4142	2.4142	9.3653
*u*_*n*_ = 3*u*_*n*−1_ + *u*_*n*−2_	3.3028	3.3028	24.8500
*u*_*n*_ = 4*u*_*n*−1_ + *u*_*n*−2_	4.2361	4.2361	50.3460
*u*_*n*_ = 5*u*_*n*−1_ + *u*_*n*−2_	5.1926	5.1926	87.1630

**Table 2:** 

*u*_*n*_	*α*	*d*	log⁡_*α*/*a*_1__ *αd*
*u*_*n*_ = 2*u*_*n*−1_ + *u*_*n*−2_ + *u*_*n*−3_	2.5468	1.5959	5.8020
*u*_*n*_ = 3*u*_*n*−1_ + 2*u*_*n*−2_ + *u*_*n*−3_	3.6274	1.9044	10.1772
*u*_*n*_ = 4*u*_*n*−1_ + 3*u*_*n*−2_ + 2*u*_*n*−3_	4.7246	1.5370	11.9086
*u*_*n*_ = 5*u*_*n*−1_ + 4*u*_*n*−2_ + 3*u*_*n*−3_	5.8074	1.2050	12.9970

**Table 3:** 

*u*_*n*_	*α*	*d*	log⁡_*α*/*a*_1__ *αd*
*u*_*n*_ = 2*u*_*n*−1_ + *u*_*n*−2_ + *u*_*n*−3_ + *u*_*n*−4_ + *u*_*n*−5_	2.6083	1.1855	4.2511
*u*_*n*_ = 3*u*_*n*−1_ + 3*u*_*n*−2_ + 2*u*_*n*−3_ + 2*u*_*n*−4_ + *u*_*n*−5_	3.9300	1.3189	6.0936
*u*_*n*_ = 4*u*_*n*−1_ + 3*u*_*n*−2_ + *u*_*n*−3_ + *u*_*n*−4_ + *u*_*n*−5_	4.6959	1.4156	11.8100
*u*_*n*_ = 5*u*_*n*−1_ + 3*u*_*n*−2_ + 2*u*_*n*−3_ + *u*_*n*−4_ + *u*_*n*−5_	5.6055	1.4828	18.5257
